# Supplementary material for: 12/111phiA Prophage Domestication Is Associated with Autoaggregation and Increased Ability to Produce Biofilm in Streptococcus agalactiae
Source: Microorganisms. 2021 May 21;9(6):1112. doi: 10.3390/microorganisms9061112 (PMC8223999; doi:10.3390/microorganisms9061112)
Supplement: Supplementary file 1 [file microorganisms-09-01112-s001.zip › microorganisms-1198640-supplementary.pdf]

microorganisms MDPI

# Supplementary files.

**Supplementary files. Table S1.** Bacterial strains used in this study.

| | Strains | N | Genotype /description | Source / reference |
|---|---|---|---|---|
| *Escherichia coli* | XL1-Blue | / | endA1 gyrA96 (Nalr ) thi-1 recA1 relA1 lac glnV44 hsdR17(rK – mK +) F= [::Tn10 (Tetr ) proAB+ lacIqZΔM15] | Stragene |
| | 12/111 (WT) | 1 | Isolated from maternal bacteraemia | [16] |
| | ΔphiA strain | 1 | Isogenic 12/111phiA deletion mutant of 12/111 | This study |
| | Δ*relB-metK* strain | 2 | Isogenic Δ*relB-metk* (BIH62_0555 to BIH62_05470) region deletion mutant of 12/111 | This study |
| | Δ*relB-yafQ* strain | 2 | Isogenic Δ*relB-yafQ* (BIH62_0555 toB1H62_05460) region deletion mutant of 12/111 | This study |
| | Δ*endonuclease-metK* strain | 2 | Isogenic Δ*endonuclease-metK* (B1H62_05465 to B1H62_05470) region deletion mutant of 12/111 | This study |
| | Δ*endonuclease* strain | 2 | Isogenic Δ*endonuclease* (B1H62_05465) deletion mutant of 12/111 | This study |
| | Δ*metK* strain | 2 | Isogenic Δ*metK* (B1H62_05470) deletion mutant of 12/111 | This study |
| | Δ*relB-metK::relB-metK* strain | 2 | *relB-metK* region in situ complementation of 12/111Δ*relB-metK* | This study |
| | Δ*relB-yafQ::relB-yafQ* strain | 2 | *relB-yafQ* region in situ complementation of 12/111Δ*relB-yafQ* | This study |
| *Streptococcus agalactiae* | Δ*endonuclease-metK ::endonuclease-metK* strain | 2 | *Endonuclease-metK* region in situ complementation of 12/111Δ*endonuclease-metK* | This study |
| | Δ*endonuclease:: endonuclease* strain | 2 | endonuclease in situ complementation of 12/111Δ*endonuclease* | This study |
| | Δ*metK ::metK* strain | 2 | metK in situ complementation of 12/111Δ*metK* | This study |
| | 12/111/ pTCV-$P_{Tet}$ | 2 | 12/111 caring empty complementation plasmid for control | This study |
| | ΔphiA /pTCV-$P_{Tet}$ | 1 | 12/111ΔphiA caring empty complementation plasmid for control | This study |
| | Δ*relB-metK*/pTCV-$P_{Tet}$ | 1 | 12/111Δ*relB-metK* caring empty complementation plasmid for control | This study |
| | ΔphiA / pTCV-$P_{Tet}$ :: *relB-yafQ* strain | 1 | *relB-yafQ* plasmid complementation of 12/111ΔphiA | This study |
| | ΔphiA /pTCV-$P_{Tet}$ :: *endonuclease-metK* strain | 1 | *endonuclease-metK* plasmid complementation of 12/111ΔphiA | This study |
| | ΔphiA /pTCV-$P_{Tet}$ :: *endonuclease* strain | 1 | *endonuclease* plasmid complementation of 12/111ΔphiA | This study |
| | ΔphiA / pTCV-PTet :: *metK* strain | 1 | *metK* plasmid complementation of 12/111ΔphiA | This study |
| | Δ*relB-metK*/pTCV-$P_{Tet}$ :: *relB-yafQ* strain | 2 | *relB-yafQ* plasmid complementation of 12/111ΔphiA | This study |
| | Δ*relB-metK*/pTCV-$P_{Tet}$ :: *endonuclease-metK* strain | 1 | *endonuclease-metK* plasmid complementation of 12/111 ΔrelB-metK | This study |
| | Δ*relB-metK*/pTCV-$P_{Tet}$ :: *endonuclease* strain | 2 | *endonuclease* plasmid complementation of 12/111 ΔrelB-metK | This study |
| | Δ*relB-metK*/pTCV-$P_{Tet}$ :: *metK* strain | 1 | *metK* plasmid complementation of 12/111Δ*relB-metK* | This study |

**Supplementary files. Table S2.** Plasmid used in this study.

| Plasmids | Genotype /description | Source / reference |
| --- | --- | --- |
| pG+host1$^{TS}$ | Replication-thermosensitive shuttle (TS) plasmid; Ery$^{r}$ | [28] |
| pTCV-P$_{Tet}$ | Mob$^{+}$ (IncP); oriR pACYC184; oriR pAM_1; complementation vector, promoter P$_{Tet}$ | [29] |
| pTCV-P$_{Tet}$ :: *relB-yafQ* | *relB-yafQ* complementation vector, promoter P$_{Tet}$ | This study |
| pTCV-P$_{Tet}$ :: *endonuclease-metK* | *Endonuclease-metK* complementation vector, promoter P$_{Tet}$ | This study |
| pTCV-P$_{Tet}$ :: *endonuclease* | *endonuclease* complementation vector, promoter P$_{Tet}$ | This study |
| pTCV-P$_{Tet}$:: *metK* | *metK* complementation vector, promoter P$_{Tet}$ | This study |

**Supplementary files. Table S3**: Primers used in this study.

| Functions | Primers | Sequence (5′–3′) |
|---|---|---|
| Deletion of *relB-metK* region | AR1 | CTTACAGGATCCATGTTCTCCTTCATCAAGCTG |
| | AR2 | TTTTTGCGTGGACTCCTACAGTTTCTTTAATTGTATC |
| | AR3 | AAACTGTAGGAGTCCACGCAAAAAGCCCAACC |
| | AR4 | GCCGTGGAATTCTTCATAATTGGCGTAGAAAGTTATTGGAAC |
| Deletion of *relB-yafQ* region | AR1 | CTTACAGGATCCATGTTCTCCTTCATCAAGCTG |
| | AR64 | GACTTTGATGAGAGTATCCTCCTACAGTTTCTTTAATTGTATC |
| | AR65 | TAGGAGGATACGCTCATCAAAGTCACTTTATG |
| | AR66 | ATTATCGAATTCAACAACTAGTAGGAGTGGTT |
| Deletion of *endonuclease-metK* region | AR58 | TCTTTGGGATCCTTGGTATTTCTGTTACTGACGC |
| | AR71 | GTGGAGGAAAACACCATAAGAAAAAAGCCACTCG |
| | AR72 | TTTTCTTATGGTGTTTTCCTCCACGCAAAAAG |
| | AR4 | GCCGTGGAATTCTTCATAATTGGCGTAGAAAGTTATTGGAAC |
| Deletion of *endonuclease* region | AR67 | TCTTTGGGATCCTTGGTATTTCTGTTACTGACGC |
| | AR68 | GGGAGCAGTAAGACCATAAGAAAAAAGCCACTCG |
| | AR69 | TTTTCTTATGGTCTTACTGCTCCCAAGGAAGA |
| | AR70 | AAAGTCGAATTCCCTTATGGCATGAGAGATGTCA |
| Deletion of *metK* region | AR61 | GGAAATGGATCCGGATTTAGAGAGTGCCACATTGAACC |
| | AR62 | GTTGGGCTTTTTGGATGCCACGTCGACCAAGCA |
| | AR63 | CGACGTGGCATCCAAAAAGCCCAACCCTTGTG |
| | AR4 | GCCGTGGAATTCTTCATAATTGGCGTAGAAAGTTATTGGAAC |
| *In situ* complementation of *relB-metK* region | AR1 | CTTACAGGATCCATGTTCTCCTTCATCAAGCTG |
| | AR4 | GCCGTGGAATTCTTCATAATTGGCGTAGAAAGTTATTGGAAC |
| *In situ* complementation of *relB-yafQ* region | AR1 | CTTACAGGATCCATGTTCTCCTTCATCAAGCTG |
| | AR66 | ATTATCGAATTCAACAACTAGTAGGAGTGGTT |
| *In situ* complementation of *endonuclease-metK* region | AR58 | TCTTTGGGATCCTTGGTATTTCTGTTACTGACGC |
| | AR4 | GCCGTGGAATTCTTCATAATTGGCGTAGAAAGTTATTGGAAC |
| *In situ* complementation of *endonuclease* | AR67 | TCTTTGGGATCCTTGGTATTTCTGTTACTGACGC |
| | AR70 | AAAGTCGAATTCCCTTATGGCATGAGAGATGTCA |
| *In situ* complementation of *metK* | AR61 | GGAAATGGATCCGGATTTAGAGAGTGCCACATTGAACC |
| | AR4 | GCCGTGGAATTCTTCATAATTGGCGTAGAAAGTTATTGGAAC |
| Plasmid complementation of *relB-yafQ* | AR33 | ATTAATGGATCCGTAGGAGGATACTAAAATGG |
| | AR34 | TAAGAACTGCAGAAAAACTTTGAGCAATTTGCTCAAAGTTTTTCACTCGCATAAAGTGACTT |
| Plasmid complementation of *endonuclease-metK* | AR85 | GGTTCTCTGCAGAAAAACTTTGAGCAATTTGCTCAAAGTTTTTCGAGTGGCTTTTTTCTTATGGT |
| | AR36 | TTGAAAGGATCCGCGTGGAGGAAAACATGATTT |
| Plasmid complementation of *endonuclease* | AR85 | GGTTCTCTGCAGAAAAACTTTGAGCAATTTGCTCAAAGTTTTTCGAGTGGCTTTTTTCTTATGGT |
| | AR86 | TTTAAGGGATCCCTTCCTTGGGAGCAGTAAGATG |
| Plasmid complementation of *metK* | AR35 | CTTACACTGCAGAAAAACTTTGAGCAATTTGCTCAAAGTTTTTTGCTTGGTCGACGTGGC |
| | AR36 | TTGAAAGGATCCGCGTGGAGGAAAACATGATTT |

**Supplementary files. Table S4**: Comparison of prophagic RelB, YafQ and Metk proteins with their bacterial homologs. NS corresponding to no significant similarity found.

| Query | Subject (reference) | query cover (in %) | identity ( in %) |
|---|---|---|---|
| 12/111phiA_RelB | E.coli_RelB [45] | 48 | 26.7 |
| | E.coli_DinJ [45] | 58 | 29.6 |
| | P307_RelB [45] | NS | NS |
| | H.i._RelB [45] | 66 | 25.8 |
| | V.c._RelB [45] | NS | NS |
| 12/111phiA_YafQ | E.coli_RelE [45] | NS | NS |
| | E.coli_YoeB [45] | 44 | 32.5 |
| | aRelE [45] | NS | NS |
| | E.coli_YafQ [45] | 93 | 39.5 |
| 12/111phiA_MetK | 12/111_MetK | 97 | 44.3 |
| | E.coli_MetK1 [47] | 97 | 55.2 |
| | E.coli_MetK [47] | 95 | 56.6 |

**Supplementary files. Table S5**: Search for *S. agalactiae* strains carrying prophages similar to 12/111phiA using blastn analysis from the NCBI database (Per. Query cover >60% and %Identity >90%).

| Description | Query Cover | Per. Ident | E-value | Hit Lengh (bp) | Hit ac. numbers |
|---|---|---|---|---|---|
| Streptococcus agalactiae CUGBS591 | 97% | 99,05% | 0 | 2227680 | CP021862 |
| Streptococcus agalactiae FDAARGOS_670 | 97% | 99,04% | 0 | 2210718 | CP044090 |
| Streptococcus agalactiae Sag153 | 97% | 99,04% | 0 | 2174504 | CP036376 |
| Streptococcus agalactiae Sag37 | 97% | 99,04% | 0 | 2198785 | CP019978 |
| Streptococcus urinalis NCTC13766 | 97% | 95,52% | 0 | 2144000 | LR134323 |
| Streptococcus agalactiae 32790-3A | 95% | 99,05% | 0 | 2148904 | CP029561 |
| Streptococcus agalactiae B105 | 95% | 99,05% | 0 | 2273717 | CP021773 |
| Streptococcus agalactiae FDAARGOS_512 | 95% | 99,05% | 0 | 2134138 | CP033822 |
| Streptococcus agalactiae NGBS128 (phiStag1) | 95% | 99,05% | 0 | 2074179 | CP012480 |
| Streptococcus sp. 'group B' FDAARGOS_229 | 95% | 99,05% | 0 | 2178261 | CP020432 |
| Streptococcus agalactiae NJ1606 | 84% | 94,19% | 0 | 2136438 | CP026084 |
| Streptococcus agalactiae YZ1605 | 84% | 94,06% | 0 | 2281602 | CP026082 |
| Streptococcus agalactiae C001 | 83% | 91,83% | 0 | 2121372 | CP008813 |
| Streptococcus MR1-Z1-201 | 78% | 93,41% | 0 | 41765 | LR134293 |
| Streptococcus constellatus FDAARGOS_1015 | 75% | 93,03% | 0 | 2038583 | CP066055 |
| Streptococcus constellatus FDAARGOS_1208 | 75% | 93,04% | 0 | 1978680 | CP069558 |
| Streptococcus constellatus subsp. pharyngis C1050 | 75% | 93,04% | 0 | 1991156 | CP003859 |
| Streptococcus constellatus subsp. pharyngis C232 | 75% | 93,04% | 0 | 1935414 | CP003800 |
| Streptococcus constellatus subsp. pharyngis C818 | 75% | 93,04% | 0 | 1935662 | CP003840 |
| Streptococcus phage Javan639 | 73% | 94,51% | 0 | 38797 | MK448825 |
| Streptococcus phage Javan95 | 73% | 94,50% | 0 | 38797 | MK448836 |
| Streptococcus canis HL_77_2 | 72% | 90,13% | 0 | 2152128 | CP053790 |
| Streptococcus phage Javan101 | 67% | 93,04% | 0 | 37952 | MK448666 |
| Streptococcus phage Javan107 | 67% | 93,04% | 0 | 37952 | MK448668 |
| Streptococcus phage Javan113 | 67% | 93,04% | 0 | 37901 | MK448670 |
| Streptococcus phage Javan93 | 67% | 94,42% | 0 | 37554 | MK448835 |
| Streptococcus phage Javan25 | 65% | 98,93% | 0 | 35085 | MK448717 |
| Streptococcus phage Javan32 | 65% | 98,93% | 0 | 35085 | MK448906 |
| Streptococcus phage Javan38 | 65% | 98,93% | 0 | 35085 | MK448922 |
| Streptococcus phage Javan65 | 65% | 98,93% | 0 | 35085 | MK448827 |
| Streptococcus equi subsp. zooepidemicus ATCC 35246 | 63% | 92,06% | 0 | 2167264 | CP002904 |
| Streptococcus equi subsp. zooepidemicus OH-71905 | 63% | 92,06% | 0 | 2189155 | CP046040 |
| Streptococcus equi subsp. zooepidemicus TN-714097 | 63% | 92,06% | 0 | 2188654 | CP046042 |

**Supplementary files. Table S6**: Microbial count after 24h culture in TH/MRS.

| Strains | CFU/mL | Standard Deviation |
|---|---|---|
| 12/111 | $3.8x10^5$ | $8.6x10^4$ |
| 12/111ΔphiA | $8.0x10^4$ | $5.9x10^4$ |

**Supplementary files. Table S7:** Identification of non-synonymous mutations in in situ deleted and complemented mutants. This analysis was performed using the Variation analysis service from the PATRIC server. Nucleotides modification was identified by "type" column. Syn: refers to no change on the encoded protein. Nonsyn: refers to non-synonymous mutation leading to amino acid change. "Ref-nt" and "Var-nt" corresponding respectively to sequence using like reference and sequence identified as modified. "Ref_nt_pos_change" indicated nucleotide position of the change in reference genome. "Ref_aa_pos_change" corresponding to position of amino acid (aa) involved by mutation and indicated aa replacing reference aa. "Upstream feature" refers to gene located upstream to the mutated gene. "downstream feature" refers to gene located downstream to the mutated gene. "snpEff_type" indicated location and nature of mutation. "snpEff _impact" informed of impact of detected mutations.

| Samples | Contig | Type | Ref_nt | Var_nt | Ref_nt_pos_change | Ref_aa_pos_change | Gene_ID | Function | Upstream feature | Downstream feature | snpEff_type | snpEff_impact |
|---|---|---|---|---|---|---|---|---|---|---|---|---|
| **12/111Δ*relB-metK*:: *relB-metK* n°1 VS 12/111Δ*relB-metK* n°1** | 1311.3476.con.0004 | Nonsyn | taa | Caa | 253T>C | Ter85Gln ext* | fig\|1311.3476.peg.661 | ABC transporter membrane-spanning permease, Pep export, Vex1 | hypothetical protein | ABC transporter membrane-spanning permease, Pep export, Vex1 | stop_lost | HIGH |
| | 1311.3476.con.0007 | Nonsyn | taa | Caa | 328T>C | Ter110Gln ext* | fig\|1311.3476.peg.1037 | Xylulose kinase (EC 2.7.1.17) | Xylulose kinase (EC 2.7.1.17) | hypothetical protein | stop_lost | HIGH |

A- RelB

```
P307_RelB              MPNIILSDTSASVSELKKNPMATVSAGDGFPVAILNRNQPAFYCVPAELYEKMLDA----
        56
V.c._RelB              MTTRILADVAASITEFKANPMKVATSAFGAPVAVLNRNEPAFYCVPASTYEIMMDK----
        56
12/111phiA_RelB        --MAKTANINLRIEPSTKAQAESLFGSFGISV--------------TDAINIFLNTSIME
        44
E.coli_DinJ            --MAANAFVRARIDEDLKNQAADVLAGMGLTI--------------SDLVRITLTKVARE
        44
E.coli_RelB            -----MGSINLRIDDELKARSYAALEKMGVTP--------------SEALRLMLEYIADN
        41
H.i._RelB              -MALTNSSISFRTVEKTKLEAYQVIEQYGLTP--------------SQVFNMFLAQIAKT
        45


P307_RelB              -----------LDDQELVKLVAERSNQPL-----HDVDLDKYL-----------------
        83
V.c._RelB              -----------LEDLELLAIAKERLSEDS-----VSVNIDDL------------------
        82
12/111phiA_RelB        GGFPFQIKQPRYNRETELAMEEARQIMEGKVTTKSYASVSDLMADLNED-----------
        93
E.coli_DinJ            KALPFDLRE--PNQLTIQSIKNSEAGIDV----HKAKDADDLFDKLGI------------
        86
E.coli_RelB            ERLPFKQTLLSDEDAELVEIVKER-----------LRNPKPVRVTLDE------L-----
        79
H.i._RelB              RSIPVDLNYLRPNKETLAAIDELD-----------SGNAESFFIEASENYSAEEFTKRIL
        94

P307_RelB              ---- 83
V.c._RelB              ---- 82
12/111phiA_RelB        ---- 93
E.coli_DinJ            ---- 86
E.coli_RelB            ---- 79
H.i._RelB              NGGQ 98
```

B- YafQ

```
E.coli_YoeB       ---MKLIWSEESWD-DYLYWQETDKRIVKKINELIK--DTRRTPFEG-KGKPEPLKHNLS
         53
E.coli_YafQ       MIQRDIEYSGQYSK-DVKLAQKR-HKDMNKLKYLMTLLINNTLPLPA-VYKDHPLQGSWK
         57
12/111phiA_YafQ   --MLQLVTTNQFRK-DVKRAKKR-GLNLKKLEAVLDPL-QKEETLDE-KHRDHALVGNYM
         54
E.coli_RelE       -----MAYFLDFDERALKEWRKLGSTVREQ-LK-KKLVE----VLESPRIEANKLRGMPD
         49
aRelE             -----MTYRVKIHKQVVKALQSLPKAHYRRFLEFRDILE--YEPVPREKFDVIKLEGTGD
         53

E.coli_YoeB       GFWSRRITEEHRLVYAVTDDSLLIAACRYHY---------------
          84
E.coli_YafQ       GYRDAHVEPDWILIYKLTDKL--LRFERTGTHAA--LFG-------
          92
12/111phiA_YafQ   GFRECHIEPDWLLVYAIDKGQLILTASRTGSHSD--LF--------
          90
E.coli_RelE       CYKIKLRSSGYRLVYQVIDEKVVVFVISVGKRERSEVYSEAVKRIL
          95
aRelE             LDLYRARLGDYRVIYSVNWKDKVIKILKLKPRGR--AYK-------
          90
```

## C- MetK

```
12/111phiA_MetK        ----MIFTSEQVSSGHPDKLCDQISDAIVTECLKHDKNSRVAVETLIKDNQVVVAGEVST
          56
12/111_MetK            MSERKLFTSESVSEGHPDKIADQISDAILDAILEQDPDAHVAAETAVYTGSVHVFGEIST
          60
E.coli_MetK1           -MNDYLFTSESVAEGHPDKMADQISDAILDAILLQDPWGKVACECLVKTGATIVAGEIST
          59
E.coli_MetK            -MAKHLFTSESVSEGHPDKIADQISDAVLDAILEQDPKARVACETYVKTGMVLVGGEITT
          59

12/111phiA_MetK        KYYFNLEGIVKKVLEPYGM---------RDVMVTNLLGVQSPDIAQGVDKG---------
          98
12/111_MetK            TAYVDINRVVRNTIAEIGYDKAEYGFSAESVGVHPSLVEQSPDIAQGVNEALEVRGSLEQ
          120
E.coli_MetK1           HAAVDIEKIVRNTIKEIGYDHSRLGFDGNTCCVLNILGKQSANIADGIRGH---------
          110
E.coli_MetK            SAWVDIEEITRNTVREIGYVHSDMGFDANSCAVLSAIGKQSPDINQGVDRA---------
          110

12/111phiA_MetK        ------SAGDQGIMFGYATDETPEFLPLPYVLATRVLEKLTNL-----------------
          135
12/111_MetK            DPLDLIGAGDQGLMFGFAVDETPELMPLPISLAHQLVKKLTDLRKSGELTYLRPDAKSQV
          180
E.coli_MetK1           -SMEELGAGDQGITFGYACDETSELMPATLVYAHRLMERQAQLRKSQRLPFLLPDAKSQV
          169
E.coli_MetK            -DPLEQGAGDQGLMFGYATNETDVLMPAPITYAHRLVQRQAEVRKNGTLPWLRPDAKSQV
          169

12/111phiA_MetK        ------------------------------------------------------------
          135
12/111_MetK            TVEYDDNDQPIRVDAVVISTQHDPNVTNDQLHKDVIEKVINEVIPSHYLDDQTKFFINPT
          240
E.coli_MetK1           TLRYQDNR-VHSVDTVVVSTQHSPDVSLDALREAVIEEIVKPVMPSHWLTPQTRFLVNPA
          228
E.coli_MetK            TFQYDDGK-IVGIDAVVLSTQHSEEIDQKSLQEAVMEEIIKPILPAEWLTSATKFFINPT
          228

12/111phiA_MetK        -------------------------------GHPALGKDPSKVDRSAAYMARKIAKDFVR
          164
12/111_MetK            GRFVIGGPQGDSGLTGRKIIVDTYGGYSRHGGGAFSGKDATKVDRSASYAARYIAKNIVA
          300
E.coli_MetK1           GSFVIGGPVGDCGLTGRKIIVDTYGGAACHGGGAFSGKDPSKVDRSAAYAARYVAKNIVA
          288
E.coli_MetK            GRFVIGGPMGDCGLTGRKIIVDTYGGMARHGGGAFSGKDPSKVDRSAAYAARYVAKNIVA
          288

12/111phiA_MetK        EGYAKRCEVQLAYAIGVAEPVGVYVNTFGTSDYPLEQLVGVVRERYDLTPQGIIKELNLL
          224
12/111_MetK            ADLAKKVEVQLAYAIGVAQPVSVRVDTFGTGVIAEADLEAAVRQIFDLRPAGIINMLDLK
          360
E.coli_MetK1           AGLASRCEVQLGWAIGLPRPVSVRINTFGTQTVSSEALLRGVNSHFDLSVFGIITTLDLL
          348
E.coli_MetK            AGLADRCEIQVSYAIGVAEPTSIMVETFGTEKVPSEQLTLLVREFFDLRPYGLIQMLDLL
          348

12/111phiA_MetK        DVDYTKTTCLGHFTKPY--LPWEQ-------------- 246
12/111_MetK            RPIYRQTAAYGHMGRTDIDLPWERVDKVQALKDFIASK 398
E.coli_MetK1           VPRYRKTACYGHFGRDS--FPWEVTDKAALLYEDVGR- 383
E.coli_MetK            HPIYKETAAYGHFGREH--FPWEKTDKAQLLRDAAGLK 384
```

**Supplementary files. Figure S1**. Alignment of prophagic RelB, YafQ and MetK proteins with their bacterial functional homologs. A- Alignment of prophagic RelB protein with bacterial member of RelB antitoxin family described by [45].B- Alignment of prophagic YafQ protein with bacterial member of RelE toxin family described by [45] with highlighting of amino acid essential for RelE toxin activity in bold and for YafQ toxin activity in red and bold [48,49].C- Alignment of prophagic MetK protein with functional bacterial MetK [47] and 12/111 bacterial protein.

A

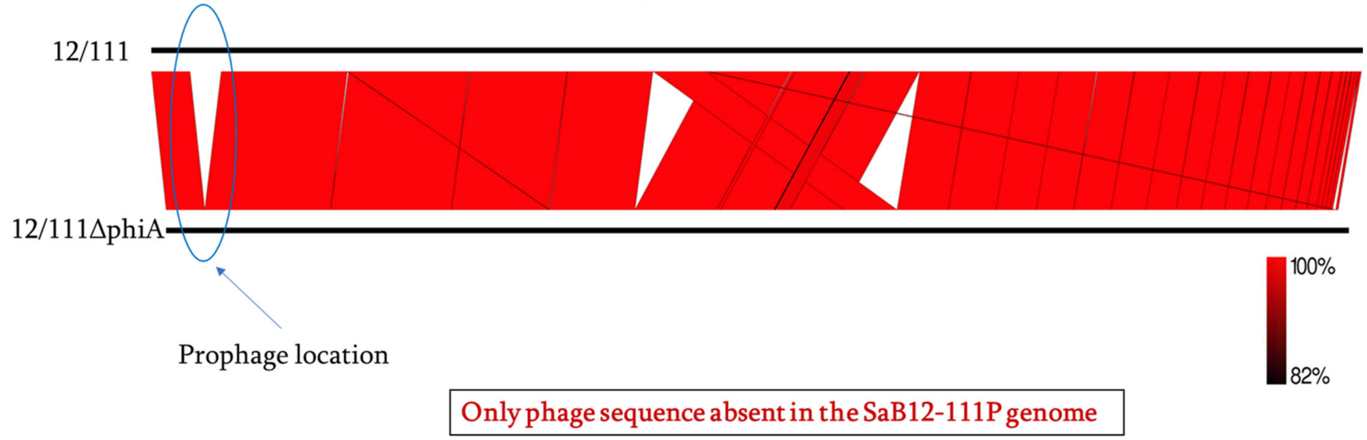


B

Pairwise Comparison of Genomic SNPs

| | 12/111ΔphiA | 12/111 |
|---|---|---|
| **12/111ΔphiA** | 0 | 0 |
| **12/111** | 0 | 0 |

**Supplementary files. Figure S2:** 12/111 vs. 12/111ΔphiA genomic comparison. A- Whole genome alignment between 12/111 and 12/111ΔphiA; deletion of the phiA from the 12/111ΔphiA genome is indicated with arow. B-Single nucleotide polymorphism (SNP) pairwise comparison.

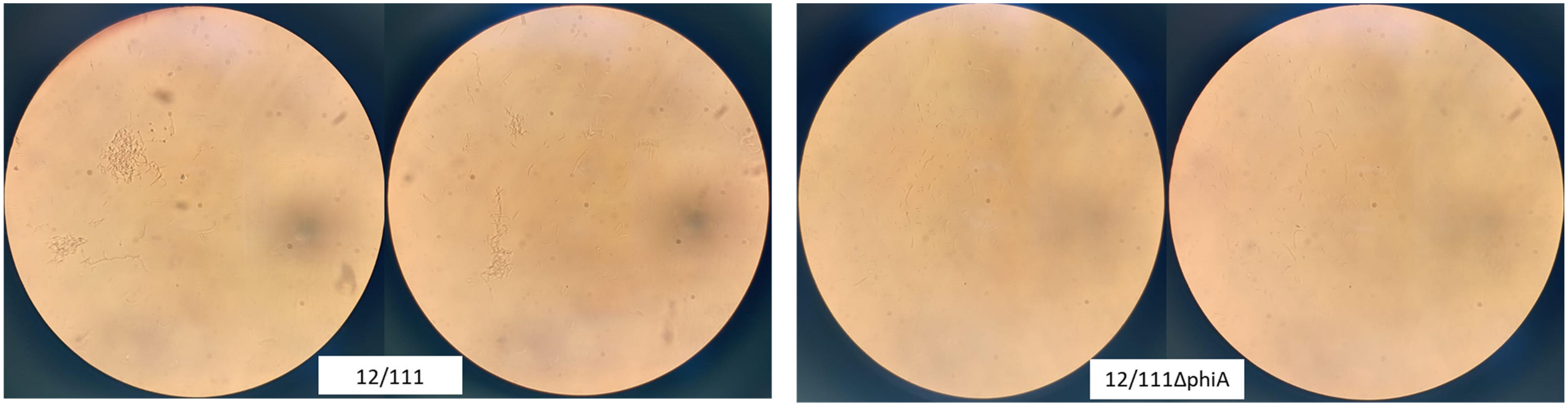


**Supplementary files. Figure S3**: Microscopic appearance of 12/111, 12/111ΔphiA. Bacterial examination by wet mount (x40).

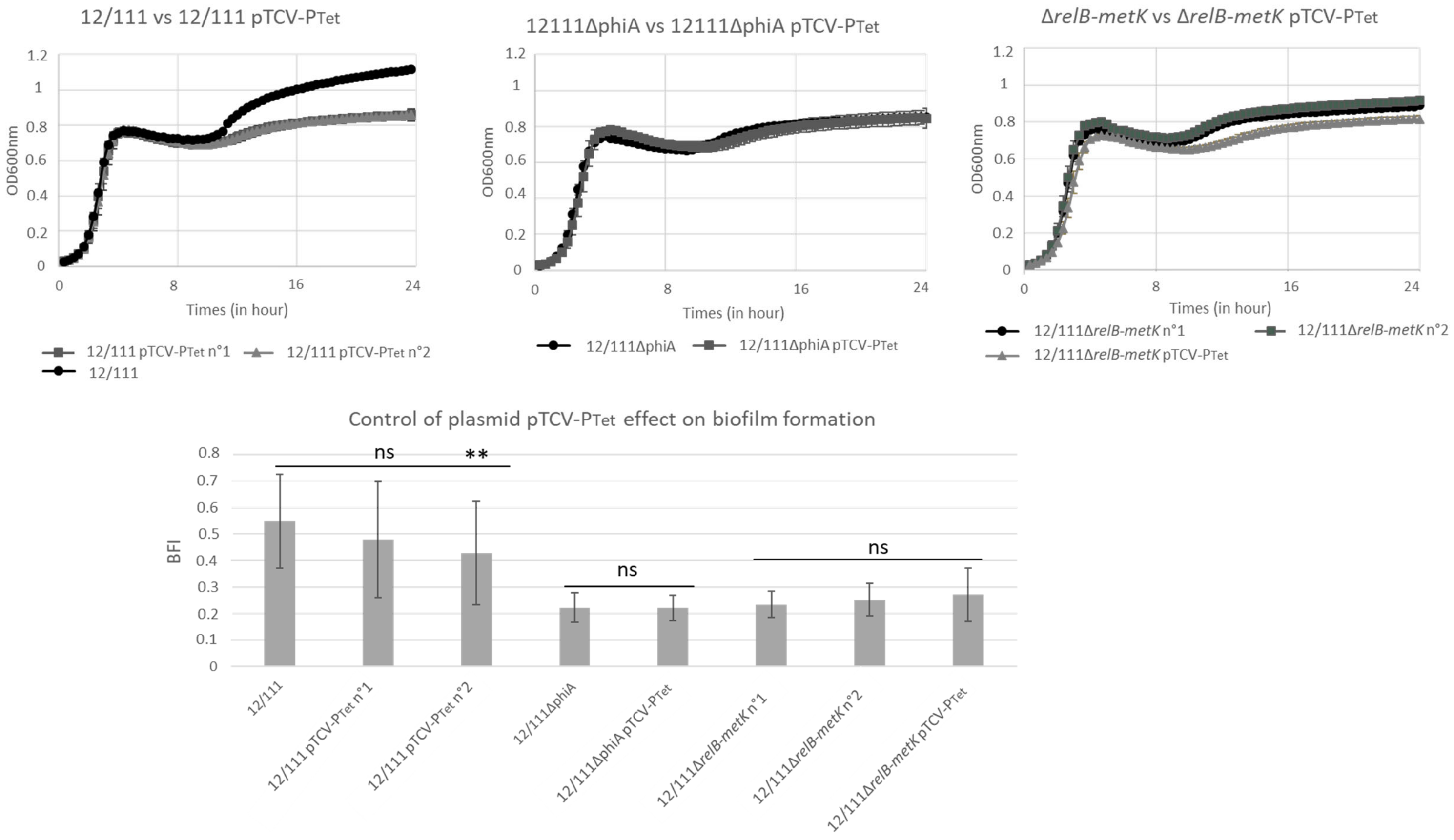


**Supplementary files. Figure S4**: Comparative growth and biofilm formation of 12/111, 12/111ΔphiA and 12/111Δ*relB-metK* respectively with 12/111 pTCV-$P_{Tet}$, 12/111ΔphiA pTCV-$P_{Tet}$ and 12/111Δ*relB-metK* pTCV-$P_{Tet}$. Growth on TH/MRS medium supplemented or not with erythromycin [10 µg/mL] at 37°C with agitation and measured every 20 min for 24h. Curve data represent mean OD600nm measurement from three independent experiments. Biofilm formation on TH 1% glucose medium and translated by biofilm formation index (BFI) value.** $p<0.01$; ns = no significance. Histogram data represent BFI mean from three independent experiments.

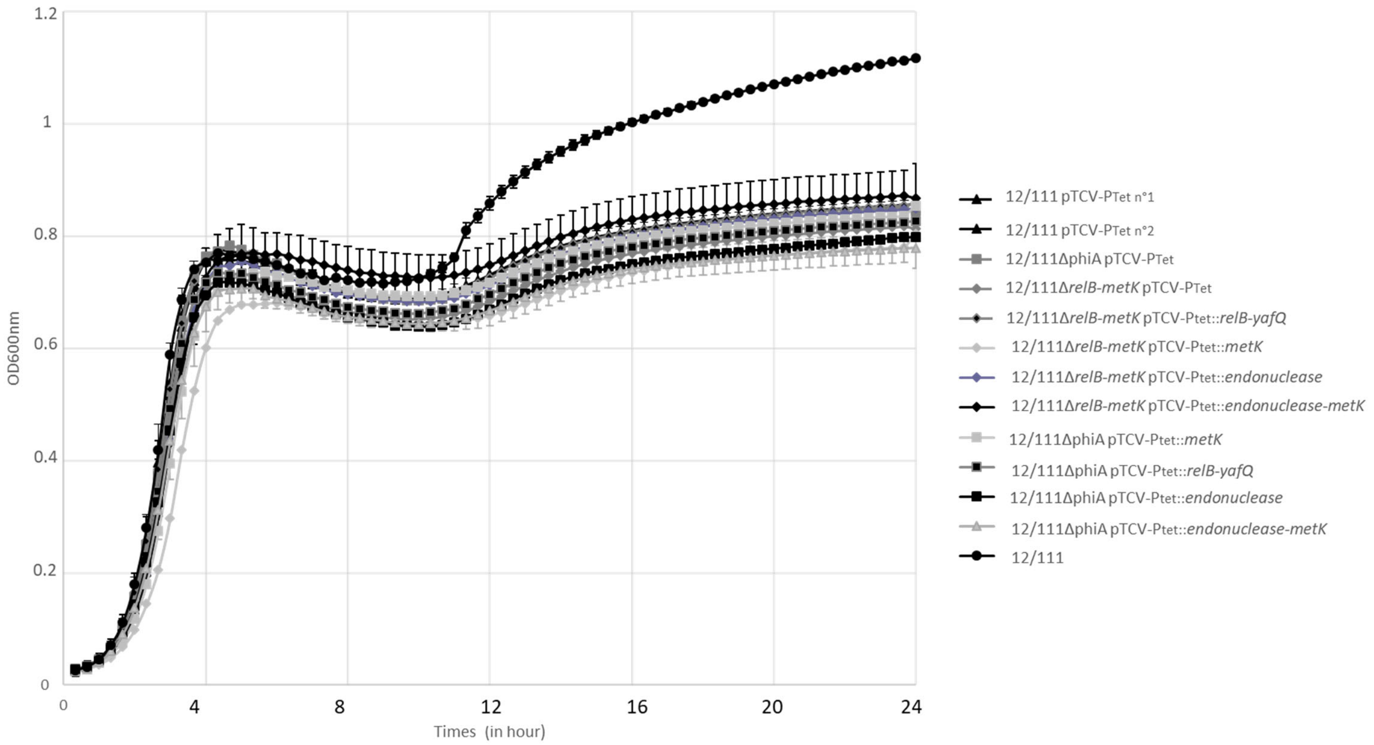


**Supplementary files. Figure S5**: Determination of *relB-yafQ*, endonuclease and *metK* ORFs involvement in *S. agalactiae* growth. Growth of 12/111 pTCV-$P_{Tet}$, 12/111ΔphiA pTCV-$P_{Tet}$, 12/111Δ*relB-metK* pTCV-$P_{Tet}$ and their associated plasmid complemented mutants. Growth on TH/MRS medium supplemented with erythromycin [10 µg/mL] (except for 12/111 control strain) at 37°C with agitation and measured every 20 min for 24h. Data represent mean OD600nm measurement from three independent experiments.

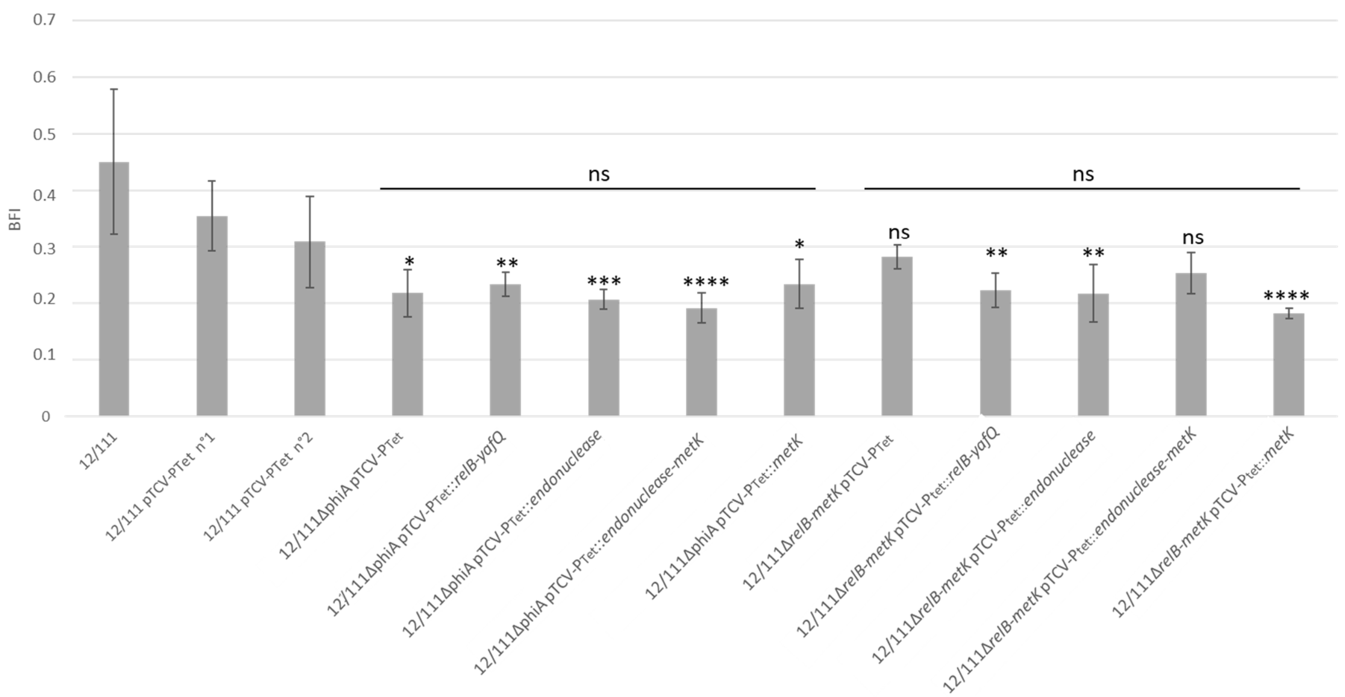


**Supplementary files. Figure S6:** Determination of *relB-yafQ*, *endonuclease* and *metK* ORFs involvement in biofilm formation. 12/111 pTCV-$P_{Tet}$, 12/111ΔphiA pTCV-$P_{Tet}$, 12/111Δ*relB-metK* pTCV-$P_{Tet}$ and their associated plasmid complemented mutants' ability to produce biofilm in TH 1% glucose translated by biofilm formation index (BFI). * corresponding p<0.05; ** to p<0.01;**** to p<0.0001; ns = no significance. Data represent mean of BFI from three independent experiment.
